# DGTR: Dynamic graph transformer for rumor detection

**DOI:** 10.3389/frma.2022.1055348

**Published:** 2023-01-11

**Authors:** Siqi Wei, Bin Wu, Aoxue Xiang, Yangfu Zhu, Chenguang Song

**Affiliations:** ^1^Beijing Key Laboratory of Intelligence Telecommunication Software and Multimedia, Beijing University of Posts and Telecommunications, Beijing, China; ^2^Faculty of Science, Beijing University of Technology, Beijing, China

**Keywords:** rumor detection, dynamic graph, transformer, rumor propagation, neural network

## Abstract

Social media rumors have the capacity to harm the public perception and the social progress. The news propagation pattern is a key clue for detecting rumors. Existing propagation-based rumor detection methods represent propagation patterns as a static graph structure. They simply consider the structure information of news distribution in social networks and disregard the temporal information. The dynamic graph is an effective modeling tool for both the structural and temporal information involved in the process of news dissemination. Existing dynamic graph representation learning approaches struggle to capture the long-range dependence of the structure and temporal sequence as well as the rich semantic association between full graph features and individual parts. We build a transformer-based dynamic graph representation learning approach for rumor identification DGTR to address the aforementioned challenges. We design a position embedding format for the graph data such that the original transformer model can be utilized for learning dynamic graph representations. The model can describe the structural long-range reliance between the dynamic graph nodes and the temporal long-range dependence between the temporal snapshots by employing a self-attention mechanism. In addition, the *CLS* token in transformer may model the rich semantic relationships between the complete graph and each subpart. Extensive experiments demonstrate the superiority of our model when compared to the state of the art.

## 1. Introduction

With the expansion of social networks, people are more likely to obtain their news through social media than traditional news sources. Social media platforms such as Twitter and Weibo facilitate the transmission and distribution of the information. However, because of the lack of adequate control and fact-checking methods for posts, they also facilitate the quick dissemination of misinformation. Rumors are false information intentionally published by users on the social media. The rumor detection task (Zhou and Zafarani, 2020) aims to identify rumors automatically based on their features.

Intuitive rumor detection methods determine the veracity of a rumor based on the content of the rumor (Yu et al., 2017; Chen et al., 2019). Content-based rumor detection approaches employ deep learning models to model news text or image content and identify rumors based on the semantic information. Although content-based rumor detection methods have produced some positive results, they suffer from some issues. First, rumors are created intentionally to confuse readers. Rumor-spreaders skillfully imitate the accurate information's lexicon, syntax, and the writing style (Ma et al., 2019). Thus, it is challenging to distinguish rumors based on their content alone. Second, a brief textual material frequently carries a rich background knowledge. Existing natural language comprehension models frequently fail to cover such a vast scope of knowledge (Dun et al., 2021), making it challenging to comprehend the exact content of news articles and resulting in a decline in performance.

Previous studies (Liu and Wu, 2018) have found that the rumor propagates differently than real news, which suggests that the propagation network of news on social networks can be used to detect rumors. It is challenging for individual users to control the propagation of rumors in social networks; consequently, propagation-based rumor detection approaches are more robust. The propagation-based approaches also do not need any additional knowledge. Due to these factors, a rising number of researchers (Ma et al., 2018; Bian et al., 2020; Lin et al., 2020; Ma and Gao, 2020) are studying the distinction between how rumors and actual facts propagate in social networks. Numerous rumor detection methods have been created depending on news propagation patterns.

Several studies (Ma et al., 2016; Chen et al., 2018; Liu and Wu, 2018; Khoo et al., 2020) have represented the process of rumor spread as a one-dimensional sequential sequence. In addition, the sequence is modeled utilizing deep neural network modeling techniques, such as RNN (Zia and Zahid, 2019), CNN (Simonyan and Zisserman, 2015), and transformer (Vaswani et al., 2017). There are certain drawbacks to the sequence structure because it just takes the sequence of information propagation into account and ignores structural information about the rumor propagation. Some studies (Bian et al., 2020; Lin et al., 2020; Silva et al., 2021) model news propagation patterns using graph structure to better capture the structure of rumor dissemination. They treat the initial news, as well as the comments and retweets, as nodes and the propagation process as edges. They turn the rumor detection problem into a graph classification problem, which got good outcomes.

**Figure 1:**
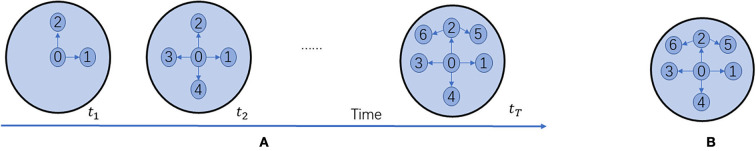
Subfigure **(A)** illustrates how to model the propagation of news using a dynamic graph structure, whereas subfigure **(B)** illustrates how to model the propagation of news using a static graph structure, where nodes signify tweets or retweets and edges denote retweets or comments. **(A)** Dynamic graph; **(B)** Static graph.

The approaches mentioned earlier all describe propagation patterns *via* static graphs. They assume that the overall structure of propagation is determined prior to the algorithm learning. All preceding methods disregard the dynamic temporal information of rumor spread. However, as shown in Figure 1, the rumor-propagation process is dynamically evolving. The dynamic evolution process of rumor propagation provides a wealth of action and time information, which can aid in our comprehension of the news propagation process and, consequently, the identification of rumors. Nonetheless, the static graph-based method disregards news transmission's dynamic and temporal information, which hinders the model's performance.

The dynamic graphs (Kazemi et al., 2020) model the entire graph as a collection of graphs under distinct time snapshots. As a result, dynamic graphs are able to accurately explain the structural and temporal information involved in the process of news propagation (Barros et al., 2021). Consequently, we intend to model the process of news spread in social networks using the dynamic graph architecture. Furthermore, we embed the structure of dynamic graphs utilizing the graph neural network approaches.

However, it is a significant problem to encode both time and structural information. The majority of the current dynamic graph-based methods (Rossi et al., 2020) use GCN to encode structural information and RNN-based methods to encode temporal information, and then use pooling methods to obtain a complete graph representation. Even though the fact that they have achieved some success, there are still specific issues. First, the GCN technique can only combine the information of first-order neighbors, and its simplistic aggregation approach ignores the semantic link between nodes. Second, it is difficult for the RNN-based temporal encoder to capture long-range relationships and is prone to gradient dispersion. Third, it is challenging for the simple pooling readout method to capture the complex semantic relationships between nodes, negatively impacting performance.

Motivated by the fact that the transformer model (Vaswani et al., 2017) efficiently captures the semantic association between parts *via* a self-attention mechanism, it has achieved excellent performance in both the natural language processing and computer vision fields. Furthermore, the special token *CLS* in the transformer captures the rich semantic association between it and each part, which can be used to characterize the overall semantics adequately. We propose transformer-based dynamic graph representation learning for rumor detection DGTR. Specifically, we employ a pretrained BERT model (Kenton and Toutanova, 2019a) to extract textual information as semantic representations of nodes from rumors and comments. We employ the row vectors of the adjacency matrix with the degree vectors of the nodes as the position embedding of the nodes. Then, the structure transformer is used to encode the structural information of the graph under each time snapshot, and the *CLS* token is used as the representation vector of the graph under that snapshot. Utilizing the temporal transformer to express the temporal relationship between the embedding vectors of each temporal snapshot, we obtain a temporal snapshot graph representation embedding with a fused temporal relationship. The classifier for the rumor detection is then fed the embeddings of each fused temporal relationship's graph representation to obtain the final classification.

In summary, our contributions are as follows:

- We consider the impact of both the structural and dynamic temporal evolution information during the dissemination process on the authenticity of the rumor. Furthermore, we propose transformer-based dynamic graph representation learning for rumor detection called DGTR. The DGTR can capture rich structurally and dynamically evolving information during news propagation, enabling improved rumor detection.
- We employ the raw transformer for learning dynamic graph representations. We create positional embedding for the graph's nodes. We utilize the structural transformer to discover the graph's structure and the temporal transformer to discover its dynamic evolution.
- Extensive experiments on two rumor detection benchmark datasets demonstrate that our proposed method achieves state-of-the-art performance.

The rest of the article is organized as follows. In Section 2, the related study is reviewed. In Section 3, we introduce the problem statement of dynamic graph-based rumor detection. In Section 4, we present the proposed method. The extensive experimental results and analysis are presented in Section 5. Finally, we conclude the article in Section 6.

## 2. Related study

The rumor detection task (Alzanin and Azmi, 2018) seeks to automatically identify rumors in social media using the news features. Depending on the news features, rumor detection tasks can be broadly categorized as either content-based or propagation-based. In this section, we briefly describe the current works for rumor detection.

### 2.1. Content-based rumor detection

Rumors in social networks commonly include text, and some also include visual information such as images or videos. The content-based rumor detection studies seek to determine a rumor's truthfulness based on its content. According on the quantity of content modalities, the content-based rumor detection approaches can be classified as single-modal methods and multi-modal methods.

#### 2.1.1. Single-modal methods

Text being the primary component of rumors, several studies on rumor detection rely on textual information. Earlier study (Agichtein et al., 2008; Song et al., 2019) employed machine learning techniques for rumor detection based on the human-specified linguistic cues. These studies are highly dependent on complex feature engineering and have limited generalization performance. Since deep neural networks can automatically extract features, they offer great modeling and generalization abilities. Numerous studies employ deep neural networks to model unimodal information for the rumor detection. Ma et al. (2016) employed recurrent neural network RNN to model the textual information of rumors, which is the first attempt at a deep neural network in the field of rumor identification. To increase the model's robustness, Ma et al. (2019) incorporated adversarial training into the RNN architecture. RNN models have difficulty modeling the long-range dependencies of text, hence Vaibhav and Hovy (2019) modeled the text with graph structures to better capture the long-range connections between words.

With the development of multimedia, the news of social networks contains not only text information but also images, videos, and other visual information that involves rich semantics. Some early studies utilize basic statistical features of the attached images such as the number of attached images (Yang et al., 2012; Wu et al., 2015), image popularity, and image type (Jin et al., 2016) to help detect rumors. For those tampered images which are digitally modified, Boididou et al. (2015) extracted advanced forensics features of image and combined them with the post-based and user-based features to detect rumors. However, these statistical features are insufficient for describing the complicated distributions of visual information in rumors. Considering deep learning approaches' high representation ability, some studies (Wang et al., 2018; Khattar et al., 2019) utilize convolutional neural networks (Simonyan and Zisserman, 2015) to mine semantic information in images to identify rumors.

#### 2.1.2. Multi-modal methods

Textual and visual features are efficacious in rumor detection tasks, respectively. It is a natural idea to combine them for better performance. Early multimodal rumor detection methods (Singhal et al., 2019, 2020) considered text and images as complementary information. They use a text encoder to extract the semantic information in the text, and an image encoder to extract the semantic information in the image, and then obtain the multimodal representation of the news through vector concatenation. However, the aforementioned methods regard image and text as complementary information, ignoring the correlation between image and text. Indeed, there are many rich semantic associations between texts and images, and the aligned parts between them usually contain crucial clues for the rumor detection. Some recent efforts (Zhang et al., 2019; Qian et al., 2021; Song et al., 2021a; Wu et al., 2021) have achieved promising results by aligning the local information of images and texts using well-designed attention mechanisms to improve information understanding the cross modalities.

Although content-based rumor detection methods can achieve good results, they are susceptible to attack from people. However, rumor authors intentionally imitate the lexical, syntactic, and writing-style features of actual news. In reality, rumor authors frequently imitate the lexical, syntactic, and writing-style characteristics of credible news to mislead the model into making incorrect decisions but current content-based approaches have difficulty in countering these artificial attacks.

### 2.2. Propagation-based rumor detection

Social psychologists have demonstrated that the process of rumor propagation in social networks differs significantly from that of real reporting. Patterns of rumor spread can be utilized as a clue to identify rumors. Earlier spread-based rumor detection methods (Liu and Wu, 2018) modeled the rumor propagation process as a linear sequence using RNN. However, the sequence simply considers the sequential information of rumor propagation and disregards the structural information. Ma et al. (2018) modeled the process of rumor propagation as a tree structure using a recurrent neural network and achieved positive results. However, they only addressed local propagation information, and it was difficult to represent global propagation information. Bian et al. (2020) employed graph structure to model rumor propagation and a graph neural network to encode it, transforming rumor detection into a graph classification task. As a result of the fact that graph topologies may model complex propagation information, good results were achieved. All the previous studies approach rumor propagation as a static structure and disregard its time-series dynamic evolution. The time-series evolution information dynamically displays the rumor propagation process in fine detail, which helps the model detect rumors.

The dynamic graph contains subgraphs at distinct time snapshots. It is able to accurately model the temporal and structural details of rumor propagation. Song et al. (2021b, 2022) proposed modeling rumor propagation patterns using dynamic graphs, using GCN to encode structural information, gating networks to encode temporal information, and average pooling of individual node embeddings to produce the full graph representation. Sun et al. (2022) leverage external knowledge to improve the model's comprehension of the text, while the way of encoding spatial and temporal information is similar with the previous methods. The GCN-based structural encoder layer can only collect first-order neighbor information, which is limited by perceptual field size and difficult to capture spatial long-range dependencies. The RNN-based temporal encoders represent sequence properties, making temporal long-range relationships challenging to capture. Simple pooling cannot model the complicated interactions between global and local information. Due to the aforementioned reasons, the existing models are inefficient. Our suggested model captures spatial and temporal long-distance relationships *via* the self-attentive mechanism of the transformer. Full graph information is represented by transformer's special *CLS* token, which contains rich global—local interaction.

## 3. Problem formulation

We translate rumor detection into a temporal subgraph classification problem on dynamic graphs. We model rumor propagation using a dynamic graph structure. A dynamic graph is defined as a series of observed snapshots $\text{𝔾}=\left\{G^{1},\ldots ,G^{T}\right\}$, where *T* is the number of time steps. Each snapshot $G^{t}=\left(V,E^{t}\right)$ is a undirected graph with a shared node set V, a link set $E^{t}$, and the weighted adjacency matrix *A*^*t*^ at time *t*. We use the structure transformer to model the association between subgraph nodes under each time snapshot *t* and the *S*−*CLS*^*t*^ token to express the embedding representation of the propagation graph $G^{t}$ under time snapshot *t*. We employ a temporal transformer to model the temporal connections between the propagation networks under each temporal snapshot of rumor propagation and use the *T*−*CLS*^*t*^ token as the final rumor representation. Finally, we train a classifier to classify the representations of rumors.

The dynamic graph-based rumor detection problem can be characterized as: given static subgraphs under each snapshot of time under the dynamic graph $\text{𝔾}=\left\{G^{1},\ldots ,G^{T}\right\}$, we want to train a classification function F:F(𝔾)→ŷ to predict the veracity of rumors.

## 4. Model

### 4.1. Model framework

**Figure 2:**
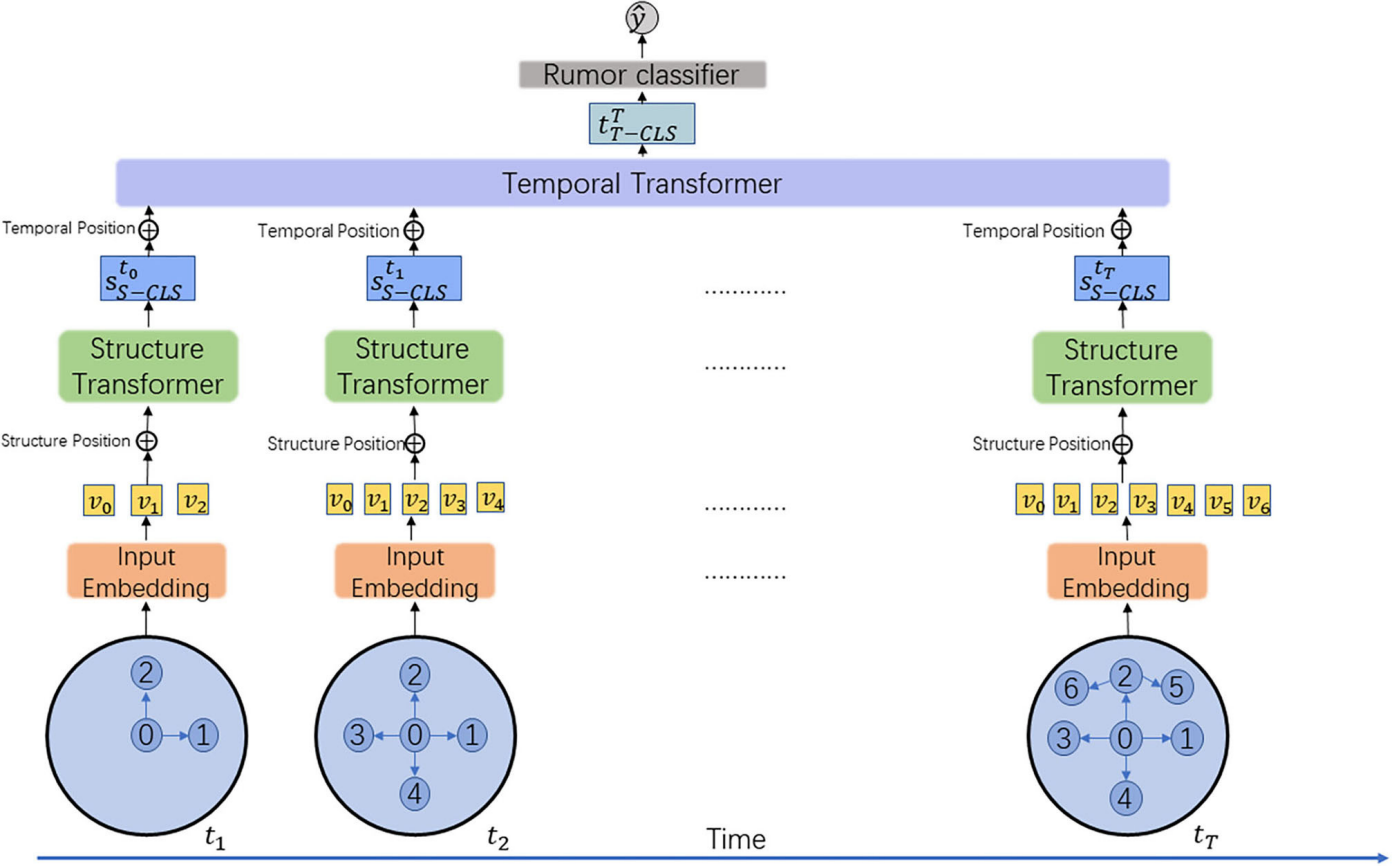
The proposed DGTR model. The **bottom** part of the figure shows the dynamic diagram of news dissemination. The **orange** part is the input embedding layer; the **green** part is the structural transformer layer; the **purple** part is the temporal transformer layer; and the **gray** part is the rumor classification layer.

The proposed framework is displayed in Figure 2. The DGTR's fundamental modules are the structural transformer component and the temporal transformer component. The input is a collection of static graph snapshots $\text{𝔾}=\left\{G^{1},\ldots ,G^{T}\right\}$ and the output is a class label ŷ. First, for a static graph snapshot $G^{t}$ derived from 𝔾, the model initially generates the raw feature representation of each node in $G^{t}$ *via* the input embedding layer. Second, the adjacency matrix and the node feature representations are fed as input to the structure transformer in order to capture the structural information of the propagation graph at this moment in time. Third, the output of the structure transformer module's propagation structure feature is fed into the temporal transformer to capture network dynamic evolutionary patterns. Finally, we leverage the dynamic evolutionary features of rumors at any time stage to detect them.

### 4.2. Input embeddings

The input embedding layer was created to encode the textual information of tweets and retweets in order to acquire initialization information for the dynamic propagation graph. This study utilizes the pre-trained BERT (Kenton and Toutanova, 2019b) to encode the text information of tweets and retweets because the pre-trained studies have demonstrated strong performance in the majority of natural language understanding tasks. It should be noted that the semantic information gained through BERT encoding is only used as the node's initialization information in the dynamic graph, which only contains the semantic information of the node itself and does not include context semantics in the propagation process. During the spread of rumors, its semantic information will change with the context. We will represent this semantic change in the subsequent dynamic graph node modeling.


(1) $$\begin{array}{l} \left[{\text{w}}_{1},\ldots ,{\text{w}}_{j},\ldots \right]\leftarrow BERT\left({\text{v}}_{i}^{t_{0}}\right), \end{array}$$

where **w**_*j*_ is the word embedding encoded by the pre-trained BERT model. We average the word embeddings in obtaining the textual information of the tweet as node **v**_*i*_'s initialization.

### 4.3. Structure transformer

The structure transformer is employed to record the structural information of the propagation graph at each time snapshot. In contrast to traditional GCN models, in which one layer can only capture information about the first-order neighbors, the transformer can model the attentional associations between all nodes and has a global understanding of the field. The traditional graph readout approach simply averages the node information to obtain a representation of the whole graph, making it difficult to model the rich semantic associations between local and global information. The transformer model designs a special *CLS* token to represent the global information and uses a self-attention mechanism to model the rich semantic associations between global and local information. We model the structural information of the temporal snapshot subgraphs using the transformer model due to these advantages.

However, the conventional transformer structure has trouble representing the graph's topological connections. To address this issue, we develop structural position embedding **sp**_*i*_ for each node to represent the position information of each node and aid the model in comprehending the topological information of the graph. Specifically, we use the corresponding row vector from the adjacency matrix to represent the nodes' structural position embedding. We use the concatenation of node semantic information and positional embedding as input to the structural transformer.


(2) $$\begin{array}{l} \begin{array}{c} {\text{s}}_{i}^{t}=\left[{\text{v}}_{i}^{t},{\text{s}\text{p}}_{i}^{t}\right], \end{array} \end{array}$$

where the [▪, ▪] operation represents vector concatenation. In addition, we define the specific [*S*−*CLS*] token to represent the propagation graph under this time snapshot.


(3) $$\begin{array}{l} \begin{array}{c} {\text{s}}_{S-CLS}^{t}\leftarrow \text{structure-transformer}\left[S-CLS,{\text{s}}_{1}^{t},\ldots ,{\text{s}}_{j}^{t},\ldots \right]. \end{array} \end{array}$$

As stated in Equation (3), we input the series of nodes with [*S*−*CLS*] token into the structure transformer for encoding and use the output ${\text{s}}_{S-CLS}^{t}$ as the global representation of the propagation graph at this time snapshot.

### 4.4. Temporal transformer

The temporal transformer is used to model the temporal association between the embeddings of the graphs under each time snapshot. Previous dynamic graph representation learning methods often use the RNN-based approaches to capture the relationships between temporal evolutions. However, RNN-based approaches have problems in capturing long-range dependencies in sequences. The transformer techniques offer good outcomes in capturing long-range dependencies in sequences through a self-attentive process. Yet, the standard transformer tends to focus on simulating the relationship between semantics, making it difficult to capture the time interval information. To solve this problem, we devise temporal position embedding **tp**^*t*^ to characterize time interval information. Specifically, we compute the time gap between the time of the retweet and the source tweet, and utilize the MLP to encode the time interval as a temporal position embedding. We concat the network information of the temporal snapshot and the temporal position embedding as the input to the temporal transformer.


(4) $$\begin{array}{l} \begin{array}{c} {\text{h}}^{t}=\left[{\text{s}}_{S-CLS}^{t},{\text{t}\text{p}}^{t}\right], \end{array} \end{array}$$

where the [▪, ▪] operation represents vector concatenation, **p^t^** indicates the timing position embedded. We design special [*T*−*CLS*] token to represent the global information of the dynamic graph. We use the temporal transformer to model the complex correlation between the local information under each temporal snapshot and the global information of the whole time phase.


(5) $$\begin{array}{l} \begin{array}{c} {\text{t}}_{T-CLS}^{T}\leftarrow \text{temporal-transformer}\left[T-CLS,{\text{h}}^{1},{\text{h}}^{2},\ldots ,{\text{h}}^{T}\right], \end{array} \end{array}$$

where the vector of time-series transformer outputs ${\text{t}}_{T-CLS}^{T}$ serves as a rumor representation of the entire time phase.

### 4.5. Rumor classification

The rumor classification layer is used to train a classifier to classify the learned rumor features ${\text{t}}_{T-CLS}^{T}$ and judge the rumor's veracity. We input the obtained rumor representations into a fully connected neural network (MLP layer) and then a softmax classifier for classification in order to obtain the classification results.


(6) $$\begin{array}{l} \begin{array}{c} ŷ=\text{softmax}\left(\text{MLP}\left({\text{t}}_{T-CLS}^{T}\right)\right), \end{array} \end{array}$$

where ŷ = [ŷ_0_, ŷ_1_] represents the model's prediction result with ŷ_0_ and ŷ_1_ indicates the predicted probability of label being 0 (non-rumor) and 1 (rumor), respectively. Because the rumor detection task is considered as a binary classification task in this study, we could define the objective functions of DGTR as follows:


(7) $$\begin{array}{l} \begin{array}{c} L\left(\Theta \right)=-y\log\left({ŷ}_{1}\right)-\left(1-y\right)\log\left({ŷ}_{0}\right), \end{array} \end{array}$$

where *y* ∈ {0, 1} denotes the ground-truth label and Θ is the learned parameters of the model.

## 5. Experiments

In this section, we conduct experiments on two real-world datasets to evaluate the effectiveness of the proposed model DGTR. Specifically, we aim to answer the following evaluation questions:

- EQ1: Is DGTR able to improve the performance of propagation-based rumor detection?
- EQ2: How do the model's various components, the structural transformer and the temporal transformer, contribute to the model's enhancement? Is it valid to use the *CLS* token to represent the global information?
- EQ3: Do the structural position embedding and temporal position embedding we designed improve the model's efficiency?
- EQ4: Can the unique *S*−*CLS* token and *T*−*CLS* token of the dynamic graph transformer effectively model the global feature of the structural and temporal domains?

In the subsequent subsections, we first describe the experimental datasets. Following that, we show a variety of state-of-the-art propagation-based rumor detection methods as baseline methods. Later, we present the model's implementation specifics. Then, we compare our methodology with existing baseline methods on two public datasets and analyze the experimental results in order to answer EQ1. Following that, we created three different sets of ablation experiments to address EQs 2, 3, and 4, respectively.

### 5.1. Datasets

In this article, we test DGTR on two widely used public benchmarks for detecting rumor, namely Weibo and FakeNewsNet. Weibo dataset is produced from Chinese Sina Weibo social media network, meanwhile FakeNewsNet dataset is constructed from Twitter social media platform. Due to the fact that all datasets include time stamps, retweet or reply relationships, and textual information, we can construct a discrete dynamic news propagation network for each item of social media news. Table 1 provides more specific statistics from two publicly available datasets.

- Weibo: This dataset was obtained by Ma et al. (2016) from one of the China's most prominent online social media platforms Sina Weibo^1^. The known rumors are collected by the Sina community management center^2^, which reports a variety of false information. Given an event, the Weibo API can capture the original message as well as all of its reposts and replies. Non-rumor events are collected by crawling general threads that are not reported as rumors. After preprocessing this dataset, we report the number of items used in this work and other details in Table 1.
- FakeNewsNet: The FakeNewsNet dataset is originally presented in Shu et al. (2020). The news articles are gathered from GossipCop^3^ and PolitiFact^4^. Using the Twitter API, the social media platform's tweets, retweets, and replies related to a news story are collected. Table 1 shows the number of items finally used in this work and more details after preprocessing this dataset.

**Table 1:** The statistics of two benchmark rumor detection datasets.

**Statistic**	**Weibo**	**FakeNewsNet**
Num of rumors	2,131	2,079
Num of non-rumors	2,207	2,089
Num of users	1,309,645	45,109
Avg. time length	1,577 h	1,951 h
Avg. num of tweets	378	42
Max. num of tweets	1,999	1,315
Min. num of tweets	10	3

Similar to previous study (Song et al., 2022; Sun et al., 2022), the source tweet, retweets, and replies are considered nodes. We consider retweet or respond behaviors as edges. The timestamps of retweets and replies are regarded as the timestamps of the edges' creation.

### 5.2. Baseline approaches

- DTC (Castillo et al., 2011): A classification approach for rumor detection that is built on decision trees and makes use of a variety of manually constructed features.
- SVM-RBF (Yang et al., 2012): A approach for detecting rumors that is based on support vector machines (SVM) and uses a radial basis function (RBF) kernel. This approach employs a set of statistical features that are extracted from tweets.
- SVM-TS (Ma et al., 2015): A classifier built on a linear SVM that uses time series modeling methods to capture the temporal features.
- ML-GRU (Ma et al., 2016): An approach that uses multilayer GRU networks to model the variable-length time series of social network rumor propagation.
- CallAtRumor (Chen et al., 2018): An approach for modeling sequences of rumor propagation using an attention-based LSTM.
- StA-HiTPLAN (Khoo et al., 2020): An approach for modeling sequences of rumor spread using a transformer. It does not account for structural information on the propagation of rumors. It employs the time delay of rumor propagation as the transformer's position embedding.
- RvNN (Ma et al., 2018): A rumor detection technique based on tree-structured recursive neural networks combines the text content and propagation structure information utilizing GRU units.
- GCN (Kipf and Welling, 2017): GCN is the most frequent graph neural network model for capturing the high-order neighborhood information. This study views rumor propagation as an undirected graph. We simulate rumor propagation using GCN and classify rumors using a fully connected layer.
- GAT (Veličković et al., 2018): Graph attention networks (GAT) use attention to learn various weights for a node's neighbors. This study uses undirected graphs to describe news propagation. And it employs GAT to simulate the distribution network of news in social networks and input the embedding to the fully connected layer to detect rumor.
- BIGCN (Bian et al., 2020): A rumor detection technique based on the static graphs. It encodes rumor propagation networks for the rumor detection using bottom-up and top-down GCN.
- DGNF (Song et al., 2022): An approach for modeling rumor propagation networks using dynamic graphs. It employs GAT to represent the structural information about the rumor propagation and a self-attentive method to simulate temporal information. It employs a straightforward mean pooling approach to generate a complete graph representation as the final rumor representation.
- DDGCN w/o knowledge (Sun et al., 2022): A dynamic graph-based rumor detection work. DDGCN simulates both rumor propagation and knowledge evolution during it. To be fair, we only evaluate changes in rumor propagation, not knowledge evolution. It employs GAT to simulate rumor structure and gated recurrent neural networks to model time series.

### 5.3. Experimental setup

To simplify comparability with present work, each dataset is separated into training and test sets, containing 80 and 20% social media news, respectively. In addition, we conducted the studies using five-fold cross-validation, which is consistent with the previous study. For Weibo and FakeNewsNet datsets, we set the time points of each snapshot as [0.0, 0.5, 1.0,1.5, 2.0, 4.0, 8.0, 16.0, 32.0, 64.0, 128.0, 256.0, 512.0, 1024.0, 2048.0, and max] hours. Because we used the pre-trained Google BERT embedding to represent each token in a phrase (Kenton and Toutanova, 2019a), word vectors are d = 768. For structure transformer component, we set the number of transformer layers to 1. For temporal transformer component, we set the number of transformer layers to 1. We train DGTR with a learning rate of 1*e*^−5^. We set batchsize and epochs at 1 and 200, respectively. Two common evaluation criteria (accuracy and the F1 score) are adopted in rumor detection to assist readers comprehend the performance of the models.

### 5.4. Results and analysis

To test the validity of the proposed model, we evaluate DGTR using two public benchmark datasets in this subsection. Table 2 displays the classification performance of the baseline approach and our approach. The top models among them are highlighted in bold font. The following conclusions may be drawn from Table 2:

- Overall, our suggested model outperformed the other baseline models on the two publicly available datasets, demonstrating its validity. The DGTR model outperforms the StA-HiTPLAN model, which is also based on the transformer, due to the fact that the StA-HiTPLAN model only analyzes the temporal information of rumor propagation and disregards the structural. The DGTR surpasses the three static graph-based algorithms GCN, GAT, and BIGCN on the two public datasets, indicating that dynamic graphs that consider both the structural and temporal information during rumor propagation are highly effective in rumor identification tasks. On both datasets, the DGTR outperforms the dynamic graph-based techniques DGNF and DDGCN without knowledge. This is due to the fact that the transformer structure efficiently captures long-range dependencies in both temporal and structural information, while employing the *CLS* token in the transformer to model the whole graph information may describe the rich correlations between global and local information.
- The performance of DGTR on the two datasets is different because of the different tweet propagation patterns in the two datasets. As seen in Table 1, the tweets in the Weibo dataset have more retweets in a shorter time, yet the FakeNewsNet dataset has fewer retweets in a longer time. The Weibo dataset has more information on Twitter propagation, which can provide richer information on the propagation structure and temporal evolution. The DGTR model can effectively capture the structural and temporal evolution information of tweets propagated in social networks. The Weibo dataset is richer in propagation information, so DGTR has better results on the Weibo dataset.
- ML-GRU, CallAtRumor, and StA-HiTPLAN all represent rumor propagation as a temporal sequence. CallAtRumor beats ML-GRU because it considers the attentional relationship between tweets to capture semantic dependencies. stA-HiTPLAN outperforms ML-GRU and CallAtRumor because it employs a transformer to represent the propagation sequence of tweets in social networks and captures long-range rumor dependencies through a self-attention method.
- Dynamic graph-based approaches DGNF and DDGCN without knowledge outperform the static graph-based algorithms GCN, GAT, and BIGCN because the latter neglect temporal and dynamic evolution information.
- The graph-based approaches (GCN, GAT, and BIGCN) beat the sequence-based algorithms (ML-GRU, CallAtRumor), which indicates that the rumor structure is crucial. Sta-HiTPLAN beats some graph-based methods because it use transformer to model associations among posts. Transformer indirectly learns a semantic association structure relation through a self-attention process, which enhances performance.

**Table 2:** Performance comparisons of different methods on Weibo and FakeNewsNet datasets.

**Model**	**Weibo**		**FakeNewsNet**	
	**ACC**	**F1**	**ACC**	**F1**
DTC	0.709	0.712	0.722	0.718
SVM-RBF	0.713	0.713	0.728	0.729
SVM-TS	0.719	0.715	0.711	0.717
ML-GRU	0.833	0.829	0.811	0.808
CallAtRumor	0.842	0.843	0.813	0.811
StA-HiTPLAN	0.871	0.867	0.879	0.876
RvNN	0.863	0.861	0.828	0.829
GCN	0.866	0.865	0.876	0.872
GAT	0.867	0.867	0.878	0.877
BIGCN	0.899	0.896	0.889	0.888
DGNF	0.933	0.932	0.922	0.921
DDGCN w/o knowledge	0.923	0.921	0.916	0.919
DGTR	**0.947**	**0.946**	**0.929**	**0.932**

The highest score is highlighted in bold.

### 5.5. Ablation study

In this subsection, we design three sets of ablation experiments to test each component's contribution to the model effect, the influence of structural position embedding and temporal position embedding on the model effect, and the role of *S*−*CLS* token and *T*−*CLS* token in modeling the rich semantic association between local and global information, respectively.

First, we conduct experiments to determine the effect of major DGTR components. Specifically, we compare the DGTR to the following variations by deleting certain components from the model:

- DGTR w/o structure transformer: In this variant, the structural transformer is removed from the DGTR. In addition, we directly average pool the node embedding vectors as the graph embedding representation for this time snapshot.
- DGTR w/ structure GCN: In this variant, we replace the structural transformer in DGTR with two layers of GCN, and we use a readout layer to take the mean value of the node vector after the GCN update to create a representation of the propagation graph under this time snapshot.
- DGTR w/o temporal transformer: In this variant, the temporal transformer is removed from the DGTR. We directly use the propagation graph embedding under the last time snapshot for rumor classification.
- DGTR w/ temporal LSTM: In this version, the temporal transformer in DGTR is replaced by LSTM. The LSTM output representations are used for rumor detection.

These variants' performance is summarized in Table 3. The following conclusions can be drawn:

- DGTR w/o structure transformer exhibited a considerable drop in effect. It proves that rumor structural information is significant for rumor detection.
- The effect of DGTR w/ structure GCN decreases compared to DGTR, but is stronger than DGTR w/o structure transformer. Structure transformer's self-attention technique can capture global dependencies between nodes during propagation. The GCN can only combine local neighbor information, making it difficult to capture long-distance graph node relationships.
- DGTR w/o temporal transformer has lower performance than DGTR. This indicates that temporal evolution information is essential for rumor detection.
- DGTR w/ temporal LSTM performs weaker than DGTR but better than DGTR w/o temporal transformer. This is because temporal transformer captures long-distance dependence between temporal snapshots and models rumor propagation's dynamic evolution effectively. LSTM has difficulty in capturing long-distance temporal dependence, hence its performance is lower.

**Table 3:** Ablation study to verify the effectiveness of each component of the model.

**Model**	**Weibo**		**FakeNewsNet**	
	**ACC**	**F1**	**ACC**	**F1**
DGTR w/o structure transformer	0.913	0.915	0.899	0.898
DGTR w/ structure GCN	0.934	0.933	0.924	0.921
DGTR w/o temporal transformer	0.911	0.912	0.897	0.896
DGTR w/ temporal LSTM	0.926	0.924	0.926	0.922
DGTR	0.947	0.946	0.929	0.932

Then, we investigate the impact of structural position embedding and temporal position embedding on the model's achievement. In particular, we evaluate the effects of DGTR and its variations with structural position embedding and temporal position embedding removed, respectively. The model's variations is displayed in the following list:

- DGTR w/o structural position embedding: In this variant, the temporal location embedding vector is omitted and the textual information of the tweet is directly used as the input to the structure transformer.
- DGTR w/o temporal position embedding: In this variant, the temporal location embedding vector is deleted and the graph information from various time snapshots is used directly as the input to the temporal transformer.

Table 4 illustrates the effects of various variants. The following conclusions can be drawn from the experimental results:

- After removing structural position embedding, DGTR effect decreases. Removing structural position embedding reduces DGTR effect. Structural position embedding lets the structural transformer get node location and degree information. And these details enable the model comprehend the topology of the rumor propagation network, which is crucial in comprehending the process of rumor spread.
- DGTR's effect reduces without temporal position embedding. The temporal position embedding helps the temporal transformer figure out the duration between temporal snapshots. It lets the model understand the dynamic temporal evolution of the rumor propagation network, which is essential for comprehending the spread process.

**Table 4:** Ablation study to verify the function of position embedding.

**Model**	**Weibo**		**FakeNewsNet**	
	**ACC**	**F1**	**ACC**	**F1**
DGTR w/o structural position embeddingr	0.901	0.899	0.902	0.903
DGTR w/o temporal position embedding	0.904	0.901	0.899	0.901
DGTR	0.947	0.946	0.929	0.932

Finally, we test whether *CLS* token helps the model capture complicated local–global interactions. We replace the *S*−*CLS* token with a global representation of the structured graph by pooling nodes with in readout layer. We replace the *T*−*CLS* token with the graph representation under the temporal snapshot output by the temporal transformer. We compare the experimental results before and after the replacement. The following variations are proposed:

- DGTR w/o *S*−*CLS* token: In this variant, we do not use the *S*−*CLS* token to represent the global structure feature under this time snapshot. We get the global structure feature by mean-pooling all node embeddings on the propagation graph.
- DGTR w/o *T*−*CLS* token: In this variant, we do not use the *T*−*CLS* token to characterize global rumor propagation. We employ the output representation under the corresponding temporal snapshot created by the temporal transformer as the temporal global feature.

Table 5 compares the performance of model variants to that of DGTR. We can draw the following conclusions from the experiment:

- After replacing the *S*−*CLS* token with mean pooling, the model effect is reduced. Mean pooling cannot capture tweets' semantic relationships. The correlation between the *S*−*CLS* token and individual tweets is generated using the self-attention. In summary, *S*−*CLS* token may model global and local information relationships in terms of rumor propagation structure.
- The model's effectiveness reduces without *T*−*CLS*. Because the output of the temporal transformer only models the evolution of the corresponding time node, it is impossible to describe the global dynamic evolution within that time interval. In summary, the *T*−*CLS* token can model the association of global and local information on the temporal sequence.

**Table 5:** Ablation experiment to verify the effect of *CLS* token.

**Model**	**Weibo**		**FakeNewsNet**	
	**ACC**	**F1**	**ACC**	**F1**
DGTR w/o *S*−*CLS* token	0.921	0.919	0.912	0.913
DGTR w/o *T*−*CLS* token	0.924	0.922	0.919	0.921
DGTR	0.947	0.946	0.929	0.932

## 6. Conclusion

In this article, we use dynamic graph structure to model the rumor propagation process and thus identify rumors. We propose a dynamic graph transformer-based rumor detection model DGTR. We use the structure transformer to capture the long-range dependencies between individual tweets under time snapshots, and the temporal transformer to capture the temporal long-range dependencies between subgraphs under individual time snapshots. In addition, we model the rich semantic interactions between local and global information on structural and temporal domains by *CLS* token. We conduct experiments on two public datasets, Weibo and FakeNewsNet. The DGTR model outperforms traditional machine learning approaches (DTC, SVM-RBF, SVM-TC), propagation sequence-based methods (ML-GRU, CallAtRumor, StA-HiTPLAN), static propagation graph-based methods (RvNN, GCN, GAT, BIGCN), and dynamic graph-based methods (DGNF). This is because DGTR uses deep learning techniques to efficiently extract structural and temporal information in the rumor propagation process. Traditional machine learning algorithms, which rely on complicated feature engineering and lack generalization, make it difficult to produce positive performance. While the propagation sequence-based technique uses deep learning models to automatically mine the sequence information in rumor propagation, it has certain drawbacks in that it ignores the structural information and temporal evolution information of news propagation. Although a well-designed graph neural network is used in the static graph-based method to mine the structural information of rumor propagation, this method performs less well than the dynamic graph-based method because it ignores the information on the temporal sequence evolution in rumor transmission. DGTR beats other graph-based dynamic approaches. This is due to the fact that DGTR can more effectively capture the structural and temporal evolution information during rumor propagation due to the transformer's ability to capture the long-range dependence of structural and temporal sequence. In future study, we will explore how to model the dynamic evolution process during rumor propagation in a more fine-grained way. We will also explore how to perform early detection of rumors by dynamic graph neural networks.

## Data availability statement

The original contributions presented in the study are included in the article/supplementary material, further inquiries can be directed to the corresponding author.

## Author contributions

SW: conceptualization, methodology, software, resources, data curation, writing—original draft, writing—review, editing, and visualization. AX and CS: data curation, writing—review, and editing. YZ: software, data curation, writing—review, and editing. BW: supervision. All authors contributed to the article and approved the submitted version.

## Footnotes

^1^ http://www.weibo.com

^2^ http://service.account.weibo.com

^3^ https://www.politifact.com/

^4^ https://www.gossipcop.com/
